# A robust MPPT framework based on GWO-ANFIS controller for grid-tied EV charging stations

**DOI:** 10.1038/s41598-024-81937-3

**Published:** 2024-12-28

**Authors:** Debabrata Mazumdar, Pabitra Kumar Biswas, Chiranjit Sain, Furkan Ahmad, Luluwah Al-Fagih

**Affiliations:** 1 Department of Electrical Engineering, National Institute of Technology Mizoram, Aizawl, 796012 India; 2 Department of Electrical Engineering, Ghani Khan Choudhury Institute of Engineering & Technology, Malda, India; 3 Qatar Environment and Energy Research Institute, Hamad Bin Khalifa University, Qatar Foundation, Doha, Qatar; 4 Division of Sustainable Development, College of Science and Engineering, Hamad Bin Khalifa University, Qatar Foundation, Doha, Qatar

**Keywords:** Battery-electric vehicles, GWO-ANFIS MPPT, Partial shading condition, Stand-by battery, Electrical and electronic engineering, Energy infrastructure

## Abstract

As electric vehicles gain popularity, there has been a lot of interest in supporting their continued development with the aim of enhancing their dependability, environmental advantages, and charging efficiency. The scheduling of navigation and charging for electric vehicles is among the most well-known research topics. For optimal navigation and charging scheduling, the coupled network state between the transportation and power networks must be met; moreover, the scheduling outcomes might significantly impact these networks. To address climate challenges, relying only on fossil fuel-based infrastructure for electric car charging is insufficient. Consequently, Multi-Energy Integrated EV charging stations have emerged as a workable solution that seamlessly integrates grid power, renewable energy sources—particularly solar energy—and EV charging needs. The enhanced grey wolf optimised (GWO) ANFIS controller for Maximum Power Point Tracking (MPPT), standby battery systems, solar power, neural network-integrated grids, and sophisticated control algorithms like PID controller are all proposed in this article as energy-efficient charging terminals for electric vehicles. Moreover, authors had considered four conditional case study and with the help of MATLAB/Simulink 2018a software, the design is thoroughly examined and assessed, providing a viable route for an efficient and sustainable EV charging infrastructure.

## Introduction

The development of transport networks has always been significantly influenced by fuel. A growing number of nations across the globe are implementing carbon-neutral policies in an effort to address the problems caused by environmental concerns and climate change. The globe is becoming more and more car-dependent every day. This reality causes the transportation sector to use a lot of oil. The earth’s surface contains finite and steadily depleting conventional energy supplies, such as coal, nuclear energy, and petroleum and its byproducts. Because of this, alternative energy sources are required to supply the world’s energy needs in the future^1^. Since electric vehicles (EVs) have a minimal carbon footprint and can be powered by sustainable energy sources like solar and wind, they have been developed as a possible replacement. Over the past ten years, this has resulted in a notable amount of interest in EVs^2^. EVs have several advantages for the environment in addition to being essential for micro-grid energy management. They function similarly to portable distributed energy storage devices in that they can store energy. an item in the grid-to-vehicle (G2V) configuration. In vehicle to grid (V2G) mode, they can also provide energy to the grid by acting as a power generating unit^3^. In the transportation sector, electric vehicles appear to be the best option for protecting the environment and human lives, provided that the electricity they use is generated from sustainable resources like solar, wind, biomass etc. They all have unique qualities and standout advantages. Solar energy, and especially photovoltaic (PV) systems, have been the most popular sustainable energy resources lately due to their safety, cleanliness, minimal maintenance requirements, noiselessness, and environmental friendliness^4,5^.

Currently on the market, plug-in hybrid electric vehicles (PHEVs), fuel cell electric vehicles (FCEVs), hybrid vehicles (HEVs), and battery electric vehicles (BEVs) are the four primary categories of electric vehicles. BEVs lack traditional internal combustion engines and only use rechargeable batteries. Although PHEVs can be plugged in for charging and have larger battery packs, they still have an electric motor. Finally, hydrogen fuel cells are used by FCEVs to produce power^6^.

Due to the current global upsurge in EV usage, scientists are looking into the grid tied electrical infrastructure. Large batteries can be compared to parked electric automobiles that are connected to the grid. Substantial parking frameworks, educational institutes, and medical contexts like hospitals, can minimise the usage of grid current at the time of peak demand or when the energy provided by non-conventional resources like solar or wind turbines- is insufficient.

In order to address the high-power demand of EVs at a particular station, the current article aims to create a reliable and efficient power supply facility that must be grid-tied. Only sustainable resources, such as solar energy, may be used to generate power without causing pollution. As a result, we also need to include a solar PV system. MPPT procedures allow for the attainment of maximum power. EV chargers come in two varieties: offboard and onboard. Selecting offboard chargers typically reduces expenses and the vehicle’s carbon footprint. The limited capacity of the onboard charge to facilitate quick charging can increase the cost of EVs because energy conversion components are more expensive.

### Literature survey

Significant scientific advancements have led to the invention and improvement of electric vehicle (EV) charging infrastructure. That means that it is necessary to look into the possibility of a strong public utility that makes use of offboard chargers, perhaps serving as charging stations^7^. The DC charging station architecture provides the connected loads with conventional DC bus service using a single AC/DC stage^8,9^. Now a days, a great deal of study has focused on microgrids’ capacity to provide the service area with adequate and consistent energy^10^. A microgrid is a small collection of electrical sources and loads that can run independently of the larger, traditionally centralised grid (macro-grid) when necessary. It can also disconnect to operate in “island mode” and synchronise with it when external factors such as weather or availability demand it. Microgrids can incorporate a variety of distributed energy resources (DERs), such as sustainable energy sources like solar panels, wind turbines, and batteries, as well as traditional generators like gas turbines or diesel engines.

To ensure efficient power utilization, energy conversion, and a smooth connection with an electrical vehicle’s charging infrastructure, various DC-to-DC converters are employed^11,12^. These converters optimise power transfer and improve system performance by serving as essential middlemen between the charging system and the RES.

Researchers specifically focus on lithium-ion batteries employed in battery electric vehicles (BEVs) because of their many benefits, including simple operation, an easy manufacturing process, low maintenance requirements, etc. Lithium-ion batteries are widely used in many electric vehicle models, including Tesla, BMW, Nissan, and others^13^. Since intensive research has allowed the lithium-ion battery to reach its current state of high energy density, high cycle life, and tremendous efficiency, it is a highly advantageous technology with clear substantial advantages^14^.

These stations must be connected to the electrical grid in order to exchange energy and control access. The two primary types of charging stations are DC and AC. The main disadvantages of AC networks are addressed by using a DC grid, which eliminates the need for energy conversion and encourages efficiency and sustainability. Notably, AC charging’s requirement that EVs be charged during off-peak hours may negatively impact EV adoption. Additionally, the delicate balance between electricity output and demand may be disturbed by abrupt load spikes brought on by fast charging grid-connected automobiles. As a result, current harmonics may appear and variations in voltage, phase, and power quality may occur^15,16^. However, the use of the DC bus in lithium-ion battery charging and discharging makes it easier to incorporate renewable energy sources like solar and wind. As a result, no more power sources are required, and no more AC grid upgrades or conversion stages are required. Numerous research have been conducted in this field, including the one presented in the publication^17^, which provides an adaptive control for charging and discharging electric car batteries using an isolated bi-directional converter. There are two different ways that an electric car battery can operate: charging and discharging.

An MPPT controller boosts a PV system’s energy efficiency by tracking the maximum power point (MPP) of a PV array^18^. There are various MPPT algorithm types created for PV systems. The first kind of MPPT algorithm that has been proposed is intended for traditional MPPT methods. Within the previously indicated approach, there are two primary subsets: offline and online MPPT techniques. Particularly common in the offline ones are the fractional short circuit current (FSCC)^19^, fractional open circuit voltage (FOCV)^20^, curve fitting (CF)^21^, constant voltage (CV)^22^, and ripple correlation control (RCC)^23^. The perturb and observe algorithm (PB&O)^24^, hill climbing (HC)^25^, and incremental conductance^26^ are the most often utilised on-line MPPT algorithms.

When a PV system has multiple peaks, both the original and improved conventional MPPT algorithms can readily converge to a local maximum power point. Some of the issues with traditional MPPT procedures are being addressed by the development of artificial intelligence-based MPPT systems. The artificial neural network (ANN)^27,28^, fuzzy logic control (FLC)^29^, genetic algorithm (GA)^30^, and adaptive neuro-fuzzy inference system (ANFIS)^31^ are among them.

Meta-heuristic-based MPPT approaches are successfully used to address the drawbacks of both artificial intelligence and conventional methods like Cuckoo Search Algorithm (CUSA)^32^, Golden Eagle Optimization (GEO)^33^, Grass Hopper Optimisation^34^, Flying Squirrel Search Algorithm (FSSO)^35^, Ant Colony Optimisation (ACO)^36^, Slap Swarm optimisation (SSO)^37,38^, and Teaching-Learning based optimisation^39^. An incredibly effective method for managing nonlinear problems is the soft computing-based MPPT approach. Unfortunately, compared to prior techniques, current MPPT algorithms are more sophisticated, expensive to build, and require a precise training dataset.

A high-gain boost converter was designed in^40^ to increase voltage, which is essential for applications requiring increased photovoltaic output. The significance of this specialised converter in RESs is increased as it effectively ramps up low input voltage to higher, usable levels. In order to achieve optimal power generation from photovoltaic sources, its design ensures excellent energy conversion. High-gain boost converters are excellent at increasing voltage, but can produce excessive amounts of heat, particularly when they are carrying heavy loads. On the other hand, the converter suggested in^41^ is adaptable and has the ability to step up and step down an input voltage. However, both converters have challenging design problems and higher switching losses. The High-Gain Single-Ended Primary Inductance Converter (SEPIC) is another noteworthy topology^42^. The converter’s continuous output current control and non-isolated operation make it suitable for solar applications, despite adding more components increasing the converter’s complexity and bulk. Furthermore, by enabling continuous input and output current as well as bidirectional power flow, the high-gain Cuk converter^43^ lowers ripple in both currents. The requirement for additional magnetic components in this topology may result in higher prices.

The authors of^44^ employed three coordinated strategies for balancing wind and stored energy in PEVs using Vehicle to Grid (V2G) technology. The energy management framework for smart homes, initially described in^45–47^, consists of smart devices, a PV system, and a PEV. PV array, a storage battery, the grid, a diesel generator, and a single voltage source converter make up the power components of the EVs charging terminals employed^48^. Rezaeimozafar et al. provided an example of how to use particle swarm optimisation and the genetic algorithm to scale green energy resources integrated into electric car charging terminals^49^. An effective lightweight plug-in electric vehicle (PEV) design with a small, affordable charging infrastructure^50^. The honey badger optimisation algorithm (HBOA) is employed in the proposed study to manage energy in solar PV systems with batteries^51^. HBOA is used to manage the DC link voltage in order to schedule the batteries. Because the HBOA has superior exploration and exploitation behaviour, there is less chance of it becoming stuck in the local optima and a smaller voltage deviation. Power quality (PQ) and switching frequencies should be considered while converting DC to AC in a grid-integrated photovoltaic system. To solve this problem, the static synchronous compensator (STATCOM) is linked at the grid side. The paper proposes a Factional Order Proportional Resonant (FOPR) controller based on the Enhanced Chimp Optimisation Algorithm (ECOA) for optimal battery scheduling^52^. Through the ECOA, the FOPR’s governing parameters are optimised based on the least amount of error.

###  Gaps in literature

In this paper, authors analysed and briefly elaborated the significant problems and areas of contention raised in the survey study. Various works on soft computing applications for renewable energies provide stability and other advantages, according to theory. When interacting with multiple module interfaces, there are some factors to take into account that differ from well-established conventional practices. The approaches^19–26^ that are provided have the advantage of being easier to adopt due to their straightforward structure and lower cost. These off-line MPPT techniques, on the other hand, are less precise and are mostly used in lower power applications with stable air conditions. Additionally, the online MPPT approaches offer excellent reliability along with a straightforward framework. Nevertheless, they experience power losses due to high-magnitude oscillations at steady state^18–26^. A few improved traditional methods are recommended in order to lower the hardware requirements and increase their functionality. When a PV system has several peaks, convergence to a local maximum power point is easy for both traditional and enhanced conventional MPPT algorithms.

Artificial intelligence methods are slower and intricate than other techniques. The truth is that ANN needs enough time to properly train. Moreover, the database utilised for training has to contain all operational circumstances. Furthermore, the implementation of the MPPT algorithm based on ANNs requires the use of costly microprocessors. Consequently, the entire system’s installation costs increase. The research points out that FLC has exceptional convergence speed. The database compiled from the expert’s experience with a certain PV module in the climate of the location where the solar system is placed, however, dictates the system’s performance. The information received is then used to build the FLC rules for the MPPT algorithm.

The main characteristics of these soft computing-based algorithms are their independence from the plant model and their non-derivative nature. Among them are ACO, PSO, and GA. There is always room for improvement, as it is not possible for a single soft computing-based technique to answer for all problems.

In summary, the above discussion emphasises the necessity of an effective MPPT controller for PV-powered EV charging stations in order to track GMPP as quickly and with nominal oscillation, minimal overshoot, and ripple as possible. The installation of a grid and standby batteries ensures that the vehicles will always have a power supply. One of the objectives of the project is to bridge a recognised research gap by providing appropriate models for all EV fleets.

### Inspiration

**Fig. 1:**
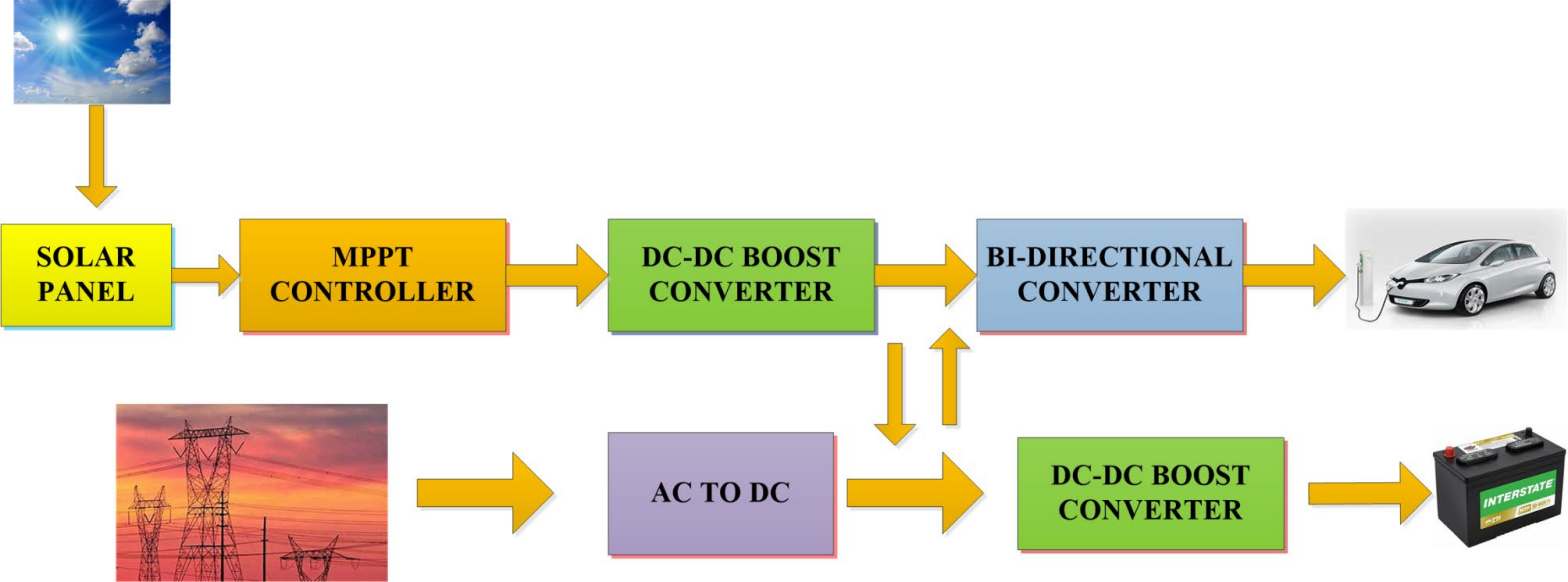
Grid-tied PV framework for EV charging terminal.

The investigators evaluated the grid as an alternative power source during their inquiry. In this study, an approach to grid-connected generating structure that employs PV systems and SBB is proposed. Electric vehicles charging infrastructure are covered in this paper comprises of a photovoltaic system controlled by an MPPT controller, an extensive examination of the numerous MPPT techniques is carried out. When the supply of photovoltaic power is limited, the standby battery pulls power from the grid and send it to the charging platforms. A 2-kW single-phase photovoltaic system that can function in both standard test settings (STC) and partial shading conditions (PSCS) is shown in this study. For duty cycle optimisation, a boost converter is used as an MPPT controller with an ANFIS algorithm tuned using the grey wolf optimisation technique. The general layout of the grid-integrated charging infrastructure for electric cars is displayed in Fig. 1.

### Achievement

The GWO proved to be an efficient solution for a variety of technological problems because of its advantages of rapid convergence, an easy-to-understand implementation structure, minimal regulating parameters, and simplicity. In light of this, a powerful and recently developed GWO-ANFIS model is provided to track MPP accurately.

An overview of this article’s primary accomplishments is provided below:

- This paper aims to design and construct a grid-tied EV charging terminal which is reliable and able to charge constantly in adverse situations. Furthermore, this research focusses on evaluating how charging station power supplies impact the scaled.
- To assess the effectiveness of developed system, the reliable performance of recommended MPPT method is examined in several parameter modifications. The recommended approach optimises the solar PV array’s output power by utilising the MPPT methodology.
- In order to meet consumer demand at charging stations, the proposed work also includes a boost converter with an MPPT scheme in grid-connected EV charging stations. When compared to other methods identified in earlier research, the created system shows good efficacy overall.

## Mathematical analysis of PV systems

**Fig. 2:**
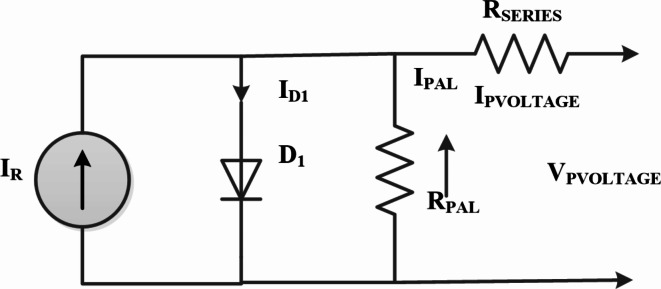
PV array configuration.

A solar PV panel is constructed from multiple interconnected photocells that operate in parallel and series to generate the necessary outputs. This is crucial to keep in mind that there are numerous PV system arrangement options^53–57^. As illustrated in Fig. 2, the most typical arrangement of a photovoltaic cell consists of a diode, a current source, and a series and parallel array of resistors. Using the Eq. (1) current produced by a solar cell is calculated.

1 $$\:{I}_{PVL}={I}_{R}-{I}_{D1}-{I}_{PAL}$$

where I_PVOLTAGE,_ V_PVOLTAGE_ individually indicate the solar cell’s output voltage and current. I_PAL_, I_D1_, I_R_ stand for parallel resistance (RPAL), diode, and photovoltaic current, respectively. R_SERIES_ stands for resistance connected in series.

2 $$\:{I}_{D1}={I}_{RSC}+{e}^{q\frac{{V}_{PVOLTAGE+}{I}_{PVOLTAGE*}{R}_{SERIES}}{nKT}\:-1}$$

The expression for current in photovoltaic cell is as outlined as Eq. (3).

3 $$\:{I}_{R}=\frac{W}{{W}_{0}}({I}_{SCN}+\lambda\:(T-{T}_{0}\left)\right)$$

4 $$\:{I}_{PVOLTAGE}={I}_{R}\:-\:{I}_{RSC\:}[{e}^{q}\frac{{V}_{PVOLTAGE+}{I}_{PVOLTAGE*\:}{R}_{SER}}{nKT}\:-1]\:-\:\frac{{V}_{PVOLTAGE}+{I}_{PVOLTAGE\:\:}*{\:R}_{SERIES}}{{R}_{PAL}}$$

**Fig. 3:**
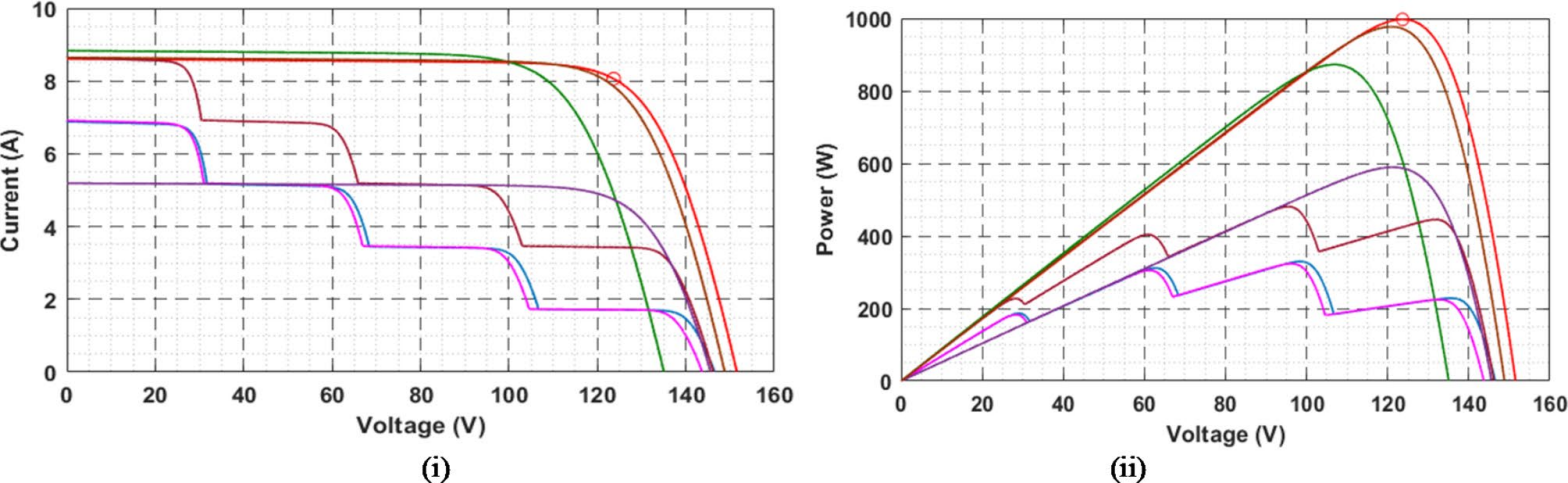
Photovoltaic array (i) I-V and (ii) P-V nature in STC and PSCS Conditions.

V-I and P-V characteristics of photovoltaic array in shown in Fig. 3. (i) and Fig. 3. (ii) in STC and PSCS conditions.

## Considered components topologies

### DC-DC boost converter

Energy from solar panels is sent to the boost converter; this energy is temperature sensitive. Heat-related effects cause PV panel power to decrease as temperature rises. This happened as a result of the open-circuit voltage of the PV array dropping with rising temperature;^58^ provides a thorough estimate of this. As a result, when temperature rises, the output power of a boost converter falls, and concurrently, the number of electron-hole pairs in the solid-state devices used in the converter increases, leading to an increase in leakage currents. Thermal protection in the converter is crucial to preventing the converter from mistakenly turning on due to regenerative action^59^. Consequently, altering the turn on time or switching frequency will control the duty ratio in one of two ways. The control of switching frequency is more intricate than the control of turn-on time. Table 1 contains information about the boost converters that are utilised here.

**Table 1:** Parameters of Boost converter.

Parameters	Value
Output capacitance (C_OUT_)	4.0704 µF
Switching frequency	10 kHz
Inductance	0.0153 H

### The bidirectional converter

The benefits of using a bidirectional boost converter are described in this work. This converter controls how electricity is stored and transferred between a solar system and a battery bank^60,61^. Compared with the other converters, the usage of a bidirectional converter is far more advantageous as per its lower cost, well-functioning ability under wide range of input output voltages, the requirement of nominal exterior components, etc. An excess amount of energy is captured, stored, and used after dusk to power an electric vehicle’s backup battery charger. The specification of the standby battery used for simulation is 240 V, 40 Ah. A minimum 20% drainage rate is estimated for standby batteries.

### Inverter module-based grid configuration

A 230 V, 50 Hz Ac grid is used to meet the additional power requirement at various charging terminals. Through an inverter this Ac grid is integrate with 500 V DC bus. A neural network designed simulation model is used that generates switching pulse for inverter relying on the percentage SOC of stand-by battery and the energy output of PV arrays.

### Battery configuration

A storage system in the form of a standby battery must be incorporated into the system in order to preserve the power supply at the charging terminals. Batteries have attracted attention among all electrical storage devices produced so far because of their high power and energy densities. While we are looking into the batteries used in EVs for charging purposes, a 7 Ah, 240 V battery is used in this article. These batteries are taking charge from a 500-volt DC bus through a DC-DC boost converter and PI controller. A minimum 10% SOC drainage rate is expected for electric vehicle batteries in this paper. The energy required for EV’s charging can be estimated by given formula:

5 $$\:{Energy}_{EV}=\:\frac{{V}_{LOW}*\:{SOC}_{E}*{Ah}_{rating}}{100}$$

Where $\:{V}_{LOW}$ is the lowest voltage of the battery, $\:{SOC}_{E}$ state of charge percentage and $\:{Ah}_{rating}$ is amp-hour rating. During the simulation operation authors assumed that internal resistance of the battery is not changing over the period of charging and discharging.

## GWO-ANFIS algorithm-based PV system framework

MPPT control techniques employed to deliver utmost power from photovoltaic system to predefined load through boost converter. Adopted method allows power flow to load, batteries or AC load systems through converter. It makes this decision by varying the boost converters on time under erratic weather conditions. In this research article, a new hybrid GWO-ANFIS MPPT controller is employed. Details of aforesaid controller are well described here in the coming section.

### Neuro-fuzzy (ANFIS) based controller

The system is based on a non-linear source, i.e., solar panel, therefore using a machine learning-based method like ANFIS would be beneficial. In order to reach the desired output value utilising a variety of input data, ANFIS is a learning method that is now commonly used. It is trained using datasets that comprise input and output parameters. ANFIS may be used in this way to solve a variety of issues. It is possible to analyse and forecast solar energy using both single and hybrid ANFIS.

**Fig. 4:**
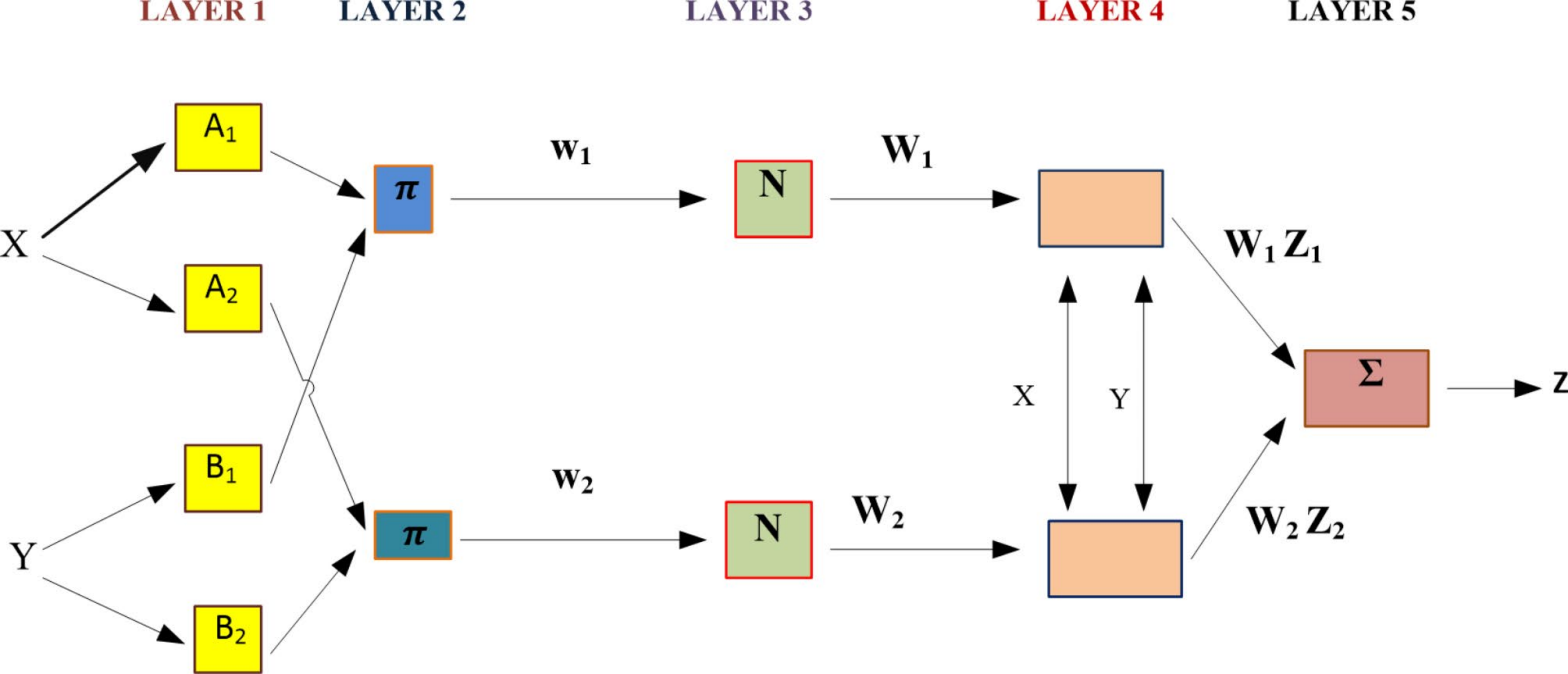
Five layers layout of ANFIS model.

Jang invented the ANFIS in the year of 1993^58^. This unique composite model makes use from fuzzy logic and neural network features both, and they work effectively together. Put another way, ANFIS is multi-layer feedforward network that is adaptive and hybrid, simulating intelligent decision-making in humans by exploiting the dual learning capabilities of FIS and ANN^60,61^. A network that mimics the behaviour of neural networks and fuzzy inference systems is referred to as an “Adaptive Neuro Fuzzy Inference System,” or ANFIS. Adaptive and non-adaptive nodes are combined in this flexible network, and synaptic weights are left out. It changes into the topology of a typical feedforward neural network. The adaptive network emulator seen in Takagi Sugeno’s adaptive fuzzy control technique is similar to the ANFIS adaptive network. In terms of operation, this adaptive network resembles a fuzzy inference system (FIS). Employing techniques like least squares, steepest descent, and backpropagation on a particular input/output dataset. There are two types of these parameters: linear and non-linear. In a fuzzy inference system, the consequent and the antecedent correspond to a rule-based system, are two essential parts of the ANFIS network. The five-layer adaptive neuro-fuzzy architecture is seen in Fig. 4. After two inputs, x and y, are received in the first layer, each node’s output is determined by the generalised Gaussian membership function (µ). These steps are represented by the following Eqs. (6)-(8)^58^. Where, $\:\mu\:$ is the Gaussian membership functions, $\:{\mu\:}_{{A}_{i}}$ and $\:{\mu\:}_{{B}_{i}}$denote the membership degrees, $\:{p}_{i}$ and ⍺_i_ are premises parameter set.

6 $$\:{O}_{1il}=\:{\mu\:}_{{A}_{i}}\left(x\right),\text{i}\hspace{0.17em}=\hspace{0.17em}\text{1,2},$$

7 $$\:{O}_{1il}=\:{\mu\:}_{{B}_{i-2}}\:\left(y\right),\text{i}\hspace{0.17em}=\hspace{0.17em}\text{3,4},$$

8 $$\:\mu\:\left(x\right)=\:\:{e}^{{-\left(\frac{x-{p}_{i}}{{a}_{i}}\right)}^{2}}$$

In the next stage, Eq. (9)^58^ is used to determine the output of every node in the second layer or the firing strength of a rule:

9 $$\:{O}_{2il}=\:{\mu\:}_{{A}_{i}}\left(x\right)\times\:\:{\mu\:}_{{B}_{i-2}}\:\left(y\right)$$

output of the third layer node is described by Eq. (10), also referred to as the normalised firing strength^56^, Where $\:{\omega\:}_{I}$ is the firing strength we get from 3rd layer.

10 $$\:{O}_{3il}={\omega\:}_{I}\:=\frac{{\omega\:}_{i}}{{\sum\:}_{i-1}^{2}{\omega\:}_{i}^{{\prime\:}}}\:\:$$

The adaptive node in the fourth layer computes its own output in the manner that follows after receiving the output $\:{O}_{3il}\:$ from the third layer, refer to Eq. (11), Where $\:{p}_{i}$, $\:{q}_{i}$ and $\:{r}_{i}\:$are the node’s consequent parameters. The solitary node at the end of the ANFIS model computes its output by the Eq. (12).

$$\:{O}_{4il}={\omega\:}_{I}{f}_{i}\:={\omega\:}_{I}({p}_{i}\text{x}+\:{q}_{i}\:\text{y}+{r}_{i}$$) (11)

12 $$\:{O}_{5}={\sum\:_{i}{f}_{i}\omega\:}_{I}\:$$

In control theory, the integrated product of time and absolute errors, or ITAE, is used as a performance metric to assess the effectiveness of control systems. It reduces steady-state errors and enhances system responsiveness in systems where it is desirable to minimize absolute error integrals over time. This criterion is frequently applied when adjusting control parameters to enhance system functionality. The ITAE technique emphasises penalising greater errors over an extended period of time and minimising errors at the first, transient reaction. Applications that call for a swift reaction and settling time must use this criterion. The following expression could be used to determine the ITAE performance index.

13 $$\:ITAE\:=\:{\int\:}_{0}^{\infty\:}t\left|e\left(t\right)\right|\:\:\:\:\:\:\:\:\:\:\:\:\:\:\:\:\:\:\:\:\:\:\:\:\:\:\:\:\:\:\:\:\:\:$$

###  GWO based ANFIS controller

**Fig. 5:**
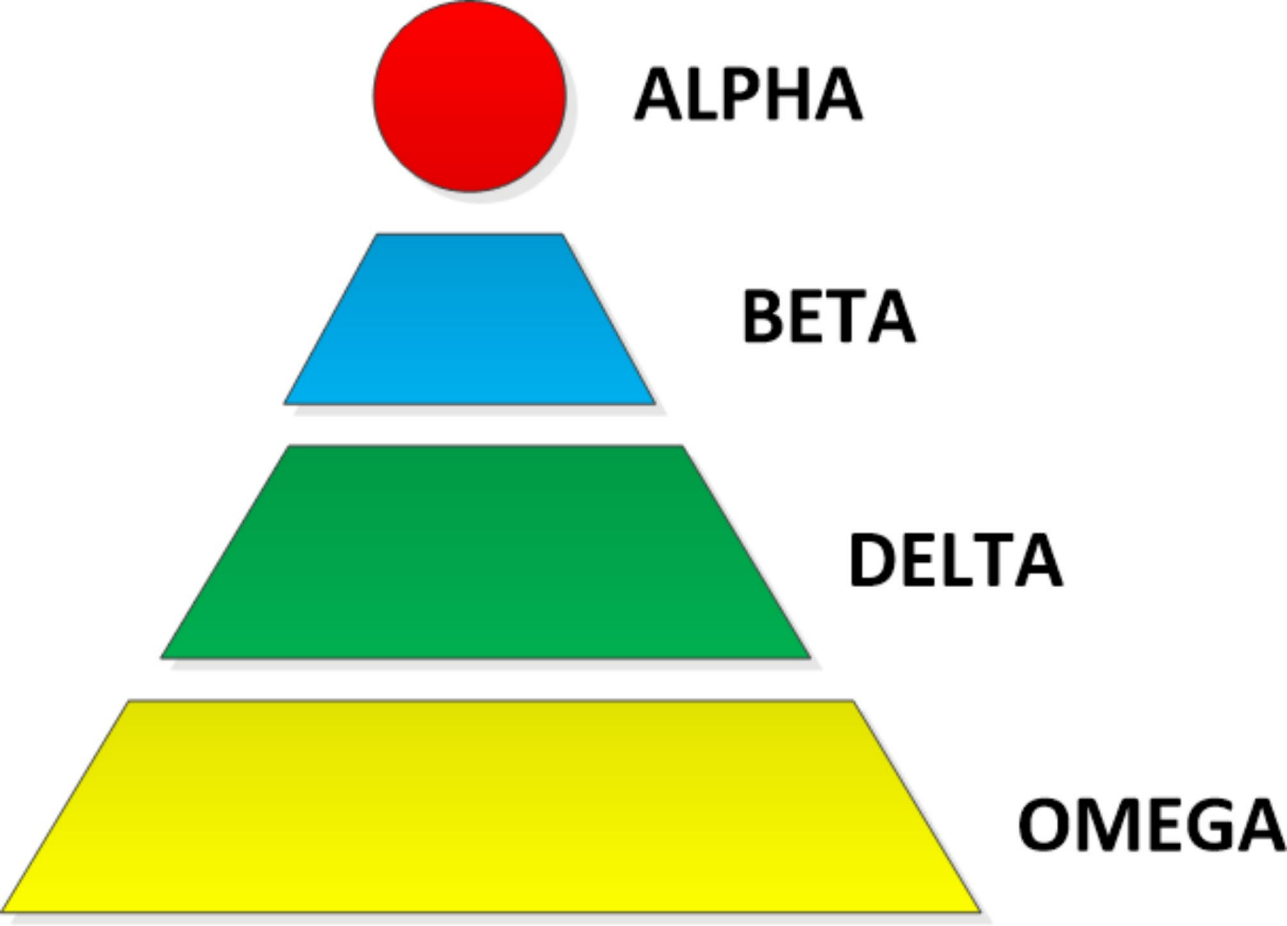
Topology hierarchy for GWO Optimisation.

In 2015, Mirjali et al. created GWO, a system that imitates the group dynamics of grey wolves^60^. Grey wolves generally chose to live in packs of five to twelve animals, and Fig. 5 depicts their rigidly hierarchical dominance hierarchy. The four groups of grey wolves—alpha, beta, delta, and omega.

The alpha wolves, who can be either male or female, are the pack leaders and are in charge of choosing where to sleep, when to wake up, when to migrate, where to hunt, and other matters. The dominant wolf, often known as the alpha wolf, is the one whom the other wolves in the pack completely obey. Only the pack’s alpha wolves are permitted to mate. Although they might not be the most physically fit members of the grey wolf group, alpha wolves are typically quite effective at leading the pack. It implies that, in contrast to their strength, the grey wolf pack’s organisation and discipline are more crucial. Located at the second rung of the hierarchy, beta wolves are able to support and aid alpha wolves in decision-making and other pack tasks. When the alpha wolf dies or ages, these beta wolves take over as pack leaders. The other lower-level wolves are under the betas’ authority in this situation, but they still owe the alpha respect. They serve as the alpha’s counsellor and enforce rules for the pack. The delta level is the subsequent level and the omega level is the last level group in the hierarchy. Although it yields to betas and alphas, the delta rules over omegas. The delta group includes scouts, hunters, elders, carers, and sentinels. Omegas are the lowest ranking wolves in the pack; hence they are always subject to the other dominant wolves. The omega wolves are the pack’s scapegoats and are the last to be let to feed.

Data on solar panel current (Ipv) and panel terminal voltage (Vpv), as well as ambient temperature (T) and solar radiation (G), were used in proposed study. The duty cycle (D) of the converter switch was also included. The input parameters in the training set used for ANFIS training were (Ipv, Vpv, G, and T), and the output parameter was the duty cycle (D), which matched these inputs. The estimated duty (ED) is quickly generated by the Fuzzy Interface System (FIS) file, which is created after training and uses the (Ipv, Vpv, G, and T) data.

### AC grid current control technique

Output from PV array in variable climate scenario and stand-by batteries SOC (%) are utilised to create a functionally adequate neural network. The fault current is found by comparing the neural network’s output current to the AC grid’s input current. The PI controller analyses the fault current, and creates a duty cycle for the inverter. A neural network’s schematic diagram is displayed in^30^.

###  Case studies consider for charging EVs

Charging stations may function in the following four conditional operation modes:

- Solar photovoltaic arrays supply the EV battery independently. The developed MPPT technique in this observation extracts the utmost power from a solar PV array while irradiance is variable, but the atmospheric temperature is not changing (25^0^C).
- Power from a solar PV panel is used to charge an electric vehicle’s battery independently. In this mode, panel is linked with selected DC bus. Maximum power can be achieved by employing the MPPT algorithm.
- Stand-by battery and solar PV panel both in operation to charge the batteries of electric vehicles. Depending on the atmospheric conditions, a hybrid model supplies the maximum amount of power.
- After sunset, the backup battery and EV battery both draw power from the AC grid when one of the input parameters—solar irradiance, for example—is not present at night.

**Fig. 6:**
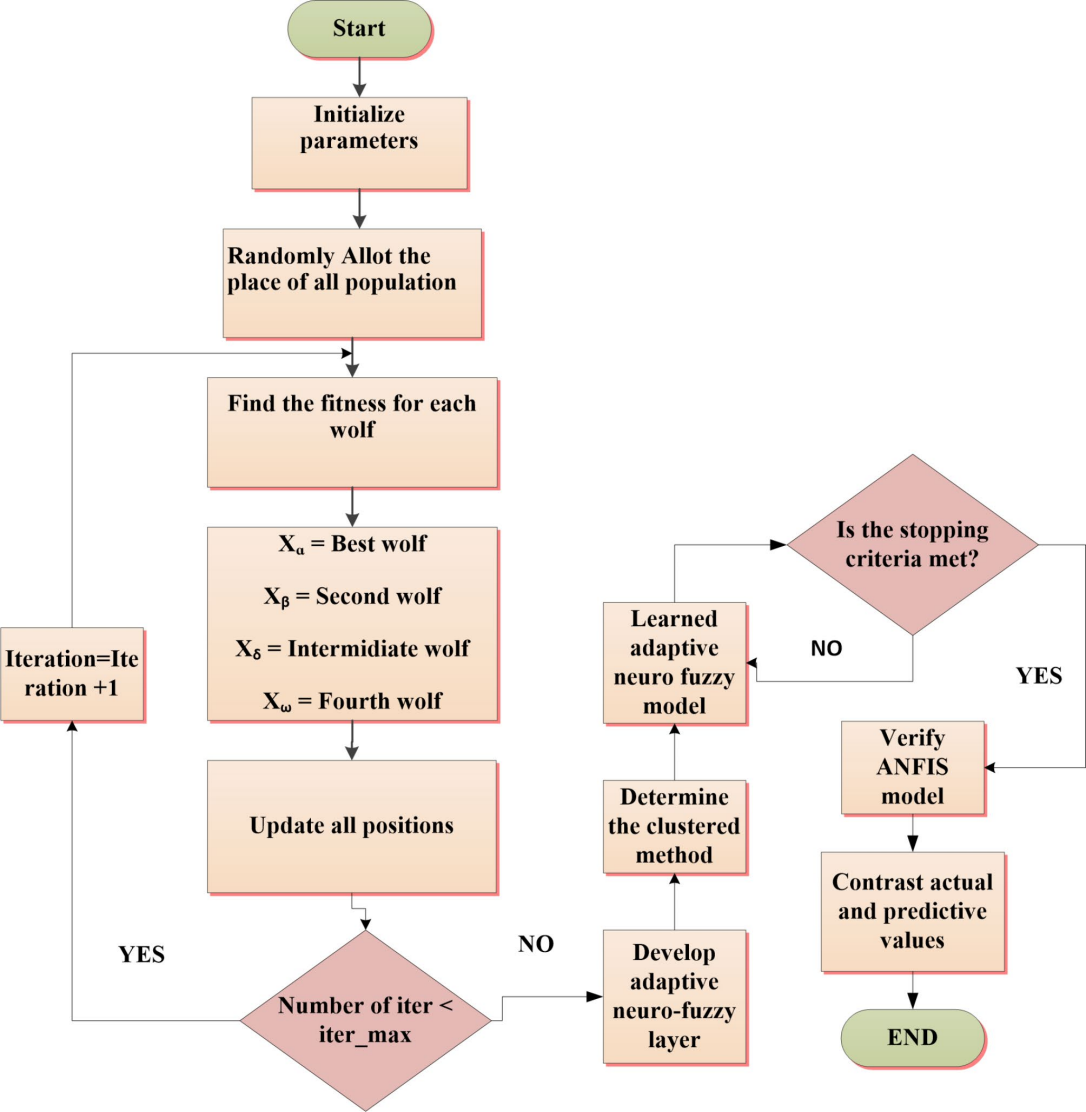
Flowchart of the developed control technique.

Figure 6 provides a detailed description of the proposed method’s flow chart.

## Simulation outcome

In this study, four different scenarios have been considered to show the supremacy of proposed method using MATLAB/Simulink. To generate the required power output, a solar array composed of eight 1Soltech 1STH-250-WH 250 W solar panels connected in series was modelled. Table 2 presents information regarding the PV system’s various parameters. The temperature coefficient for the open circuit voltage was − 0.36901 V/°C, while the maximum power output of each panel was determined as 250.205 W. This system was capable of producing sufficient voltage and current for the system’s needs due to the connection of eight modules in series per string and one parallel string.

**Table 2:** Specifications of the solar panels used in the simulation.

Parameter	Value
V_oc_	37.30 V
I_SCN_	8.66 A
Temperature coefficient at V_oc_	-0.36901 V/°C
Maximum power output	250.205 W
Series-connected modules per string	8
Parallel strings	1

It was important to note that the solar energy generated by the panels was stored in a lithium-ion battery, which was configured with a nominal voltage of 240 V and a rated capacity of 48 Ah. The lithium-ion battery was a major part of the simulation. The Table 3 summarizes the various parameters of the lithium-ion battery used in the simulation. Initially, the SOC of the battery was set at 9%, representing a scenario where the battery required recharging. To protect the battery from over-discharge, a cut-off voltage of 180 V was applied. Additionally, the battery’s response time was set at 0.0001 s to capture real-time dynamic behaviour accurately during the simulation.

**Table 3:** Parameters of the lithium-ion battery used in the simulation.

Parameter	Value
Nominal voltage	240 V
Rated capacity	48 Ah
State of charge (SOC)	9%
Cut-off voltage	180 V
Battery response time	0.0001 s

For the evaluation of the MPPT method, a variety of simulation conditions were employed, including changes in solar irradiance, temperature fluctuations, as well as varying load demands. By analyzing the response of the system to these various scenarios, it was possible to evaluate the MPPT method and its performance in a comprehensive manner. In order to conduct a detailed and accurate simulation of the interactions between solar panels and battery storage systems, MATLAB/Simulink software was used.

### Case Study 1

**Fig. 7:**
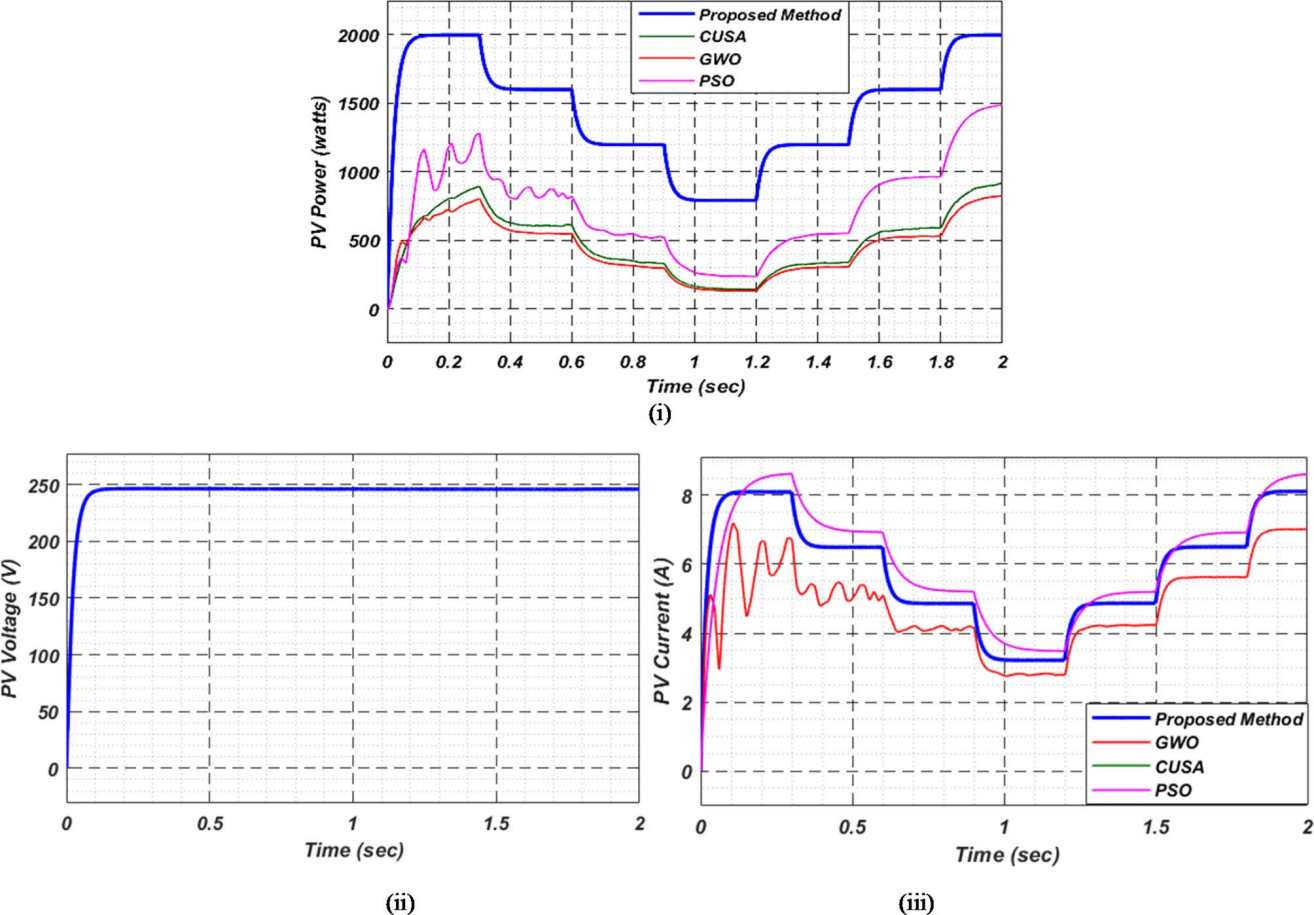
(i) PV power (ii) voltage and (iii) current for observation − 1.

**Fig. 8:**
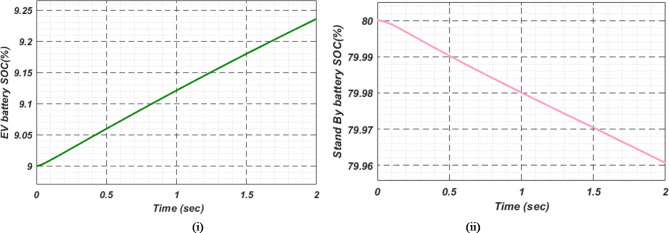
(i) Electric vehicle’s Battery and (ii) Stand-By battery’s state-of-charge (SOC) for case study 1.

**Fig. 9:**
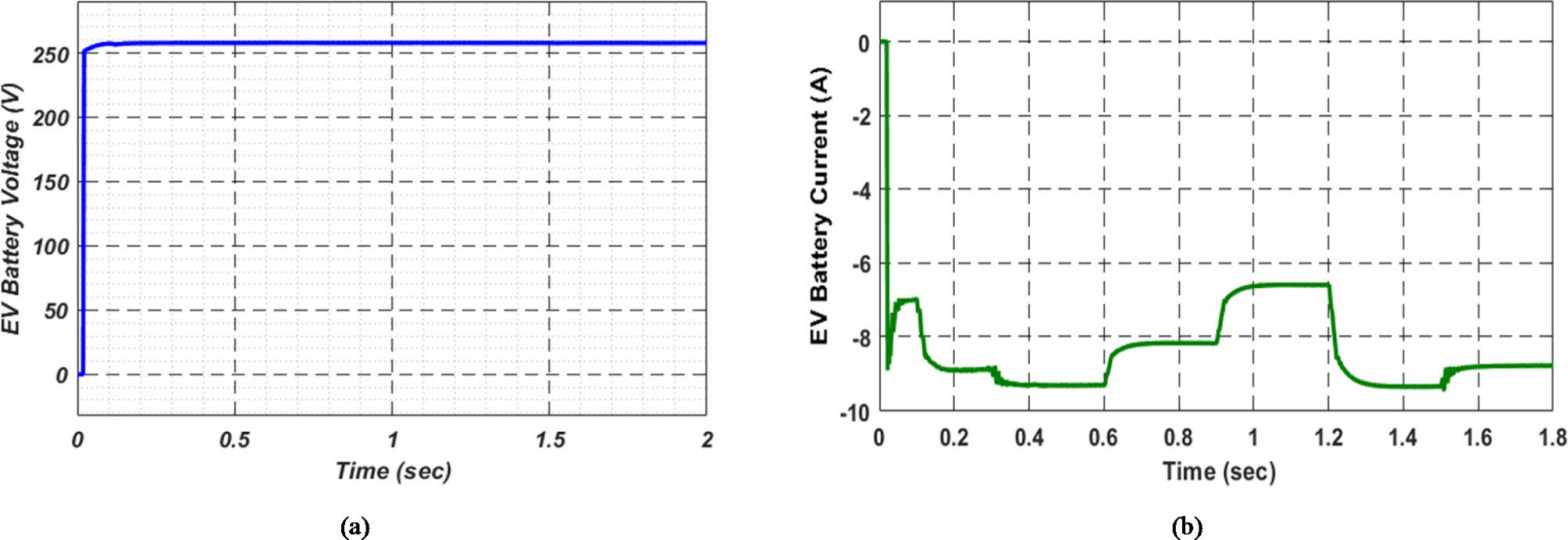
(i) Battery voltage and (ii) Battery current of EV at case study − 1.

**Fig. 10:**
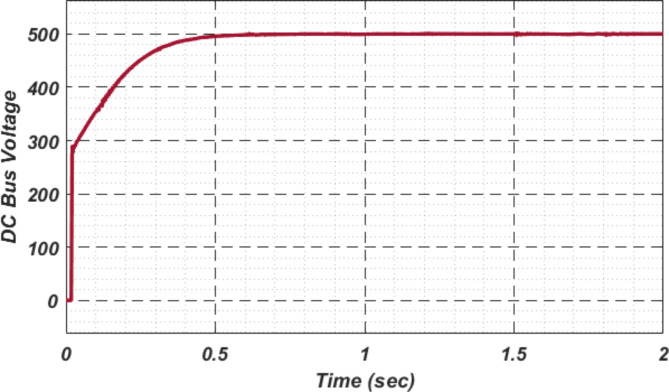
DC Bus voltage.

**Fig. 11:**
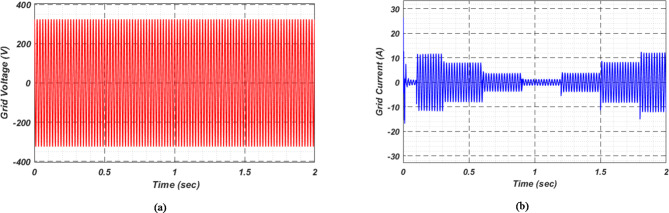
(i) Grid Voltage (ii) Grid current for scenario-1.

**Fig. 12:**
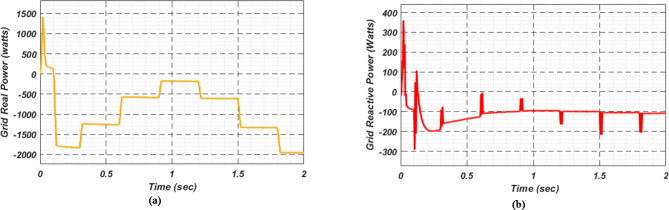
(i) Grid real (ii) Grid reactive power for scenario-1.

At first authors considered that air conditions are variable with fluctuating solar irradiation initially at a nearly constant temperature of 25^0^C. In MATLAB/SIMULINK model. During observation, total 2 sec time span is considered. In first instance, the irradiance is taken at 1000 w/m^2^. It is fluctuating, going from 1000 w/m^2^ to 800-600-400-800 w/m^2^ appropriately, every 0.3 s, and then returning to 1000 w/m^2^ after 1.8 s. PV power from solar panel may be achieved 2000 W if the voltage remains at 250 V. The photovoltaic array provides maximum power and reaches the MPP very quickly and with low settling time as seen in Fig. 7. (i). Because photovoltaic power varies in response to variations in irradiance, but PV voltage remains constant (as demonstrated in Fig. 7 (ii), the equivalent PV current is also depicted in Fig. 7 (iii). For modelling purposes, an EV battery with a SOC of 9% and a standby battery with a SOC of 80% are first taken into account shown in Fig. 8 (i) and (ii). Figure 9 (i) illustrates that the battery voltage of an electric vehicle (EV) remains constant at 250 V. Battery current is negative because the battery is currently taking continuous charge, as seen in Fig. 9 (ii). The DC bus voltage is fixed at 500 V, as shown in Fig. 10. An inverter makes it possible to connect the DC bus to the AC grid. Under the partial shadowing conditions indicated in Fig. 11, a sinusoidal voltage in this instance indicates whether we have fluctuating grid current due to fluctuations in solar irradiance. Even the uncertain atmospheric conditions impact the actual and reactive power of the AC grid, as shown in Fig. 12 (i) and (ii).

### Case Study 2

**Fig. 13:**
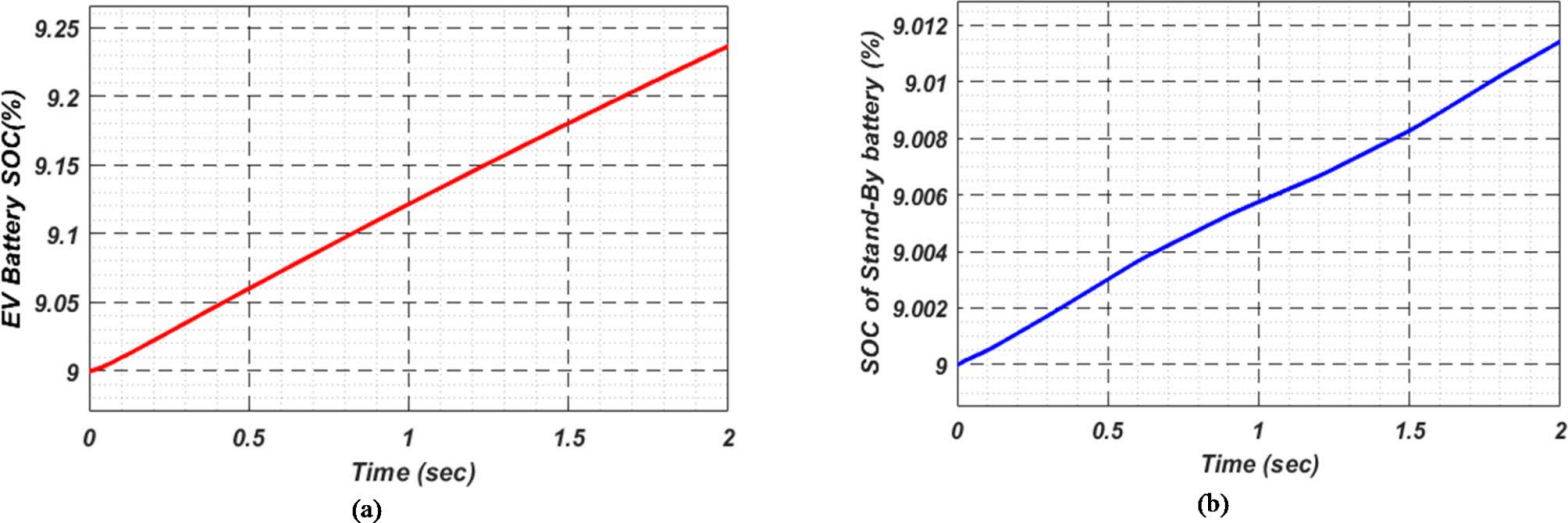
(i) EV Battery and (ii) Stand-By battery’s state-of-charge (SOC) for case study 2.

**Fig. 14:**
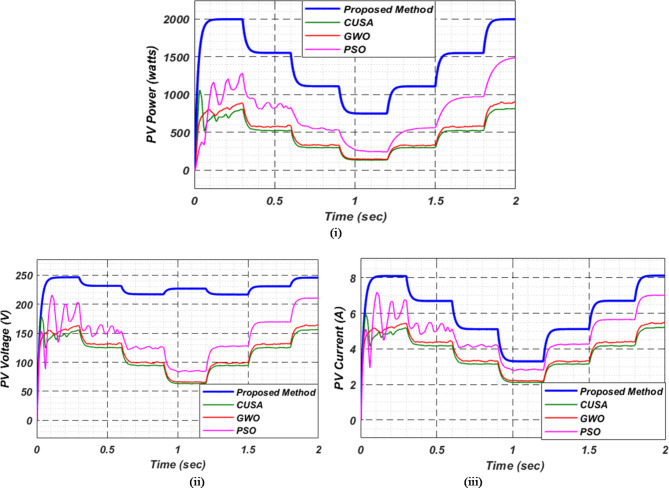
(i) Photovoltaic power (ii) PV voltage and (iii) PV current for observation-2.

**Fig. 15:**
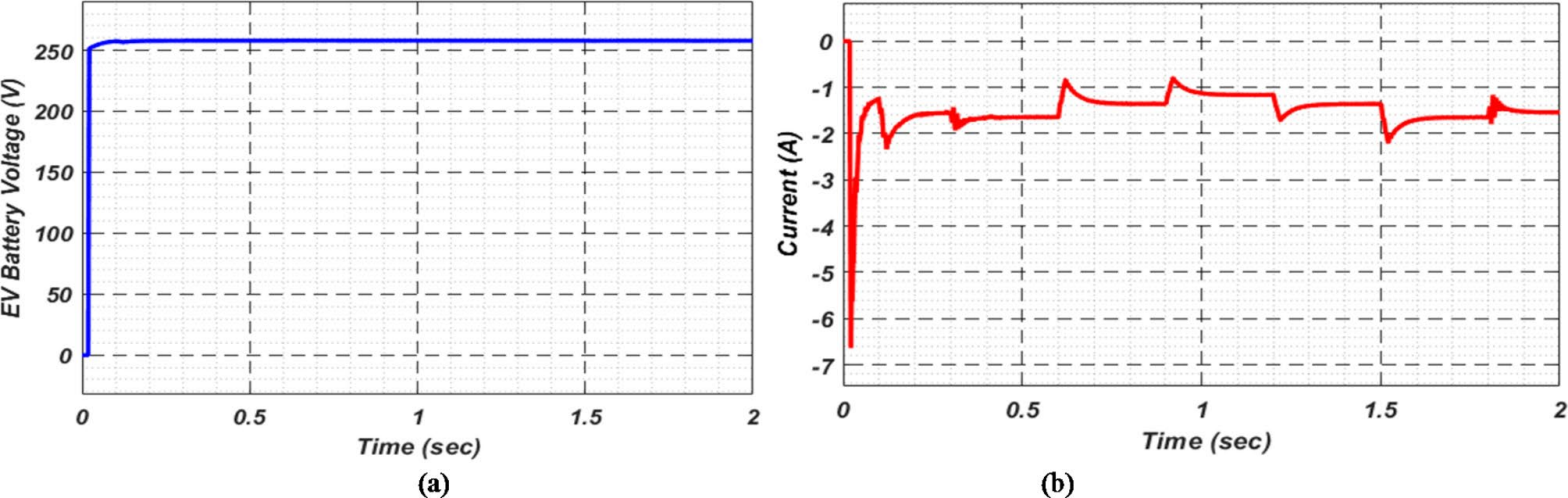
(i) Battery voltage and (ii) Battery current of EV at case study-2.

**Fig. 16:**
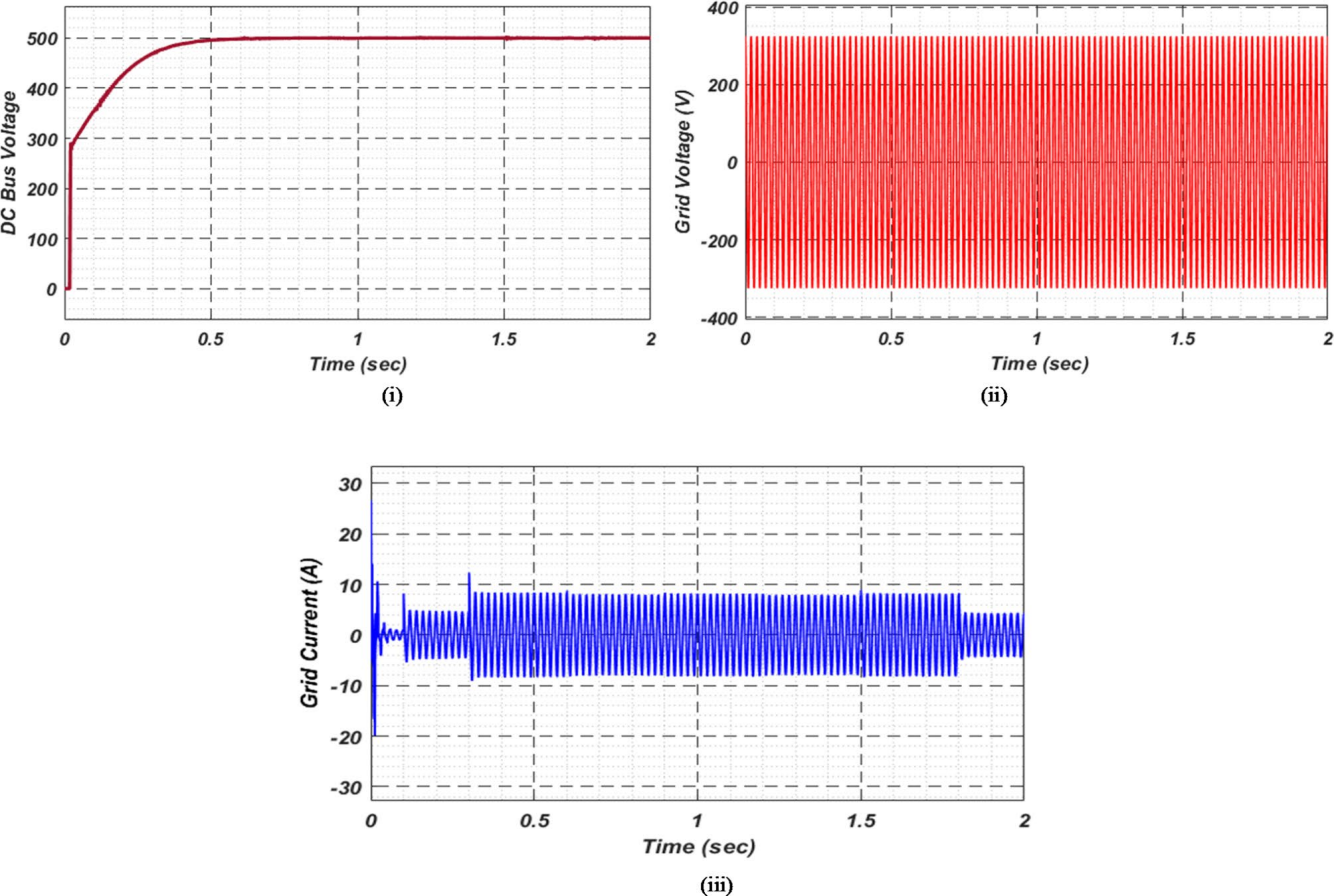
(i) DC bus voltage (ii) Grid voltage (iii) Grid current for observation − 2.

**Fig. 17:**
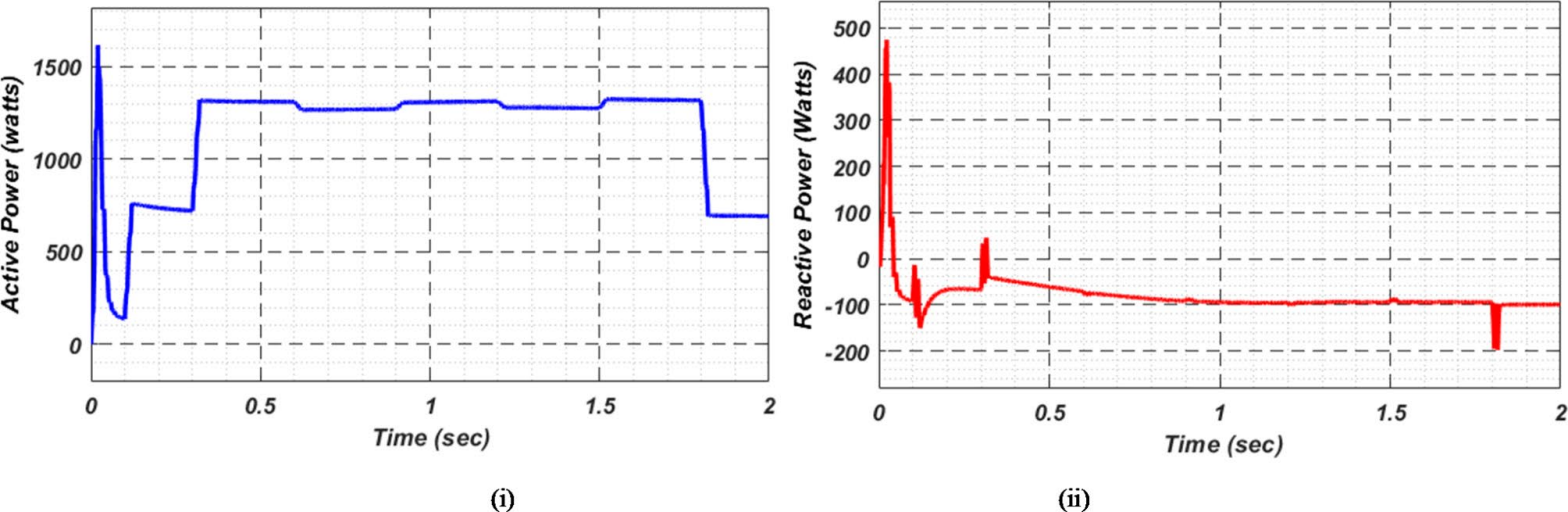
(i) Grid real and (ii) Grid reactive power for scenario-2.

Under this subsection a new assumption considered by authors. Practically with variable irradiance, atmospheric temperature also fluctuates. In this case also authors selected a time span of 2 s and after every 0.3 s not only irradiance but also temperature is varying. The temperature that goes along with the irradiance is increasing first in line with previous case; it goes from 25 °C to 37.5 °C, and then it instantly returns to 25 °C after 1.8 s. Both EVs battery and stand-by battery are taking charge in this real-time scenario, which is shown in Fig. 13. Comparison with other MPPT methods available in literature, proposed technique gives more stable and maximum power, voltage and current as shown in Fig. 14. This MPPT method has lowest settling time, lesser oscillation, and maximum power compare with other existing methods in literature. As per the charging station concern, Fig. 15. (i) shows constant EVs battery voltage which indicates a stable charging infrastructure, Fig. 15 (ii) indicates a negative EVs battery current cause battery is taking charge at that particular time. A fixed DC bus voltage of 500 V is shown in Fig. 16. (i). To establish a connection between the DC bus and the AC grid, an inverter is employed. Here, a sinusoidal voltage controls whether we receive different grid currents in response to variations in solar irradiation and air temperature when there is partial shadowing. Figure 16. (ii) and 16 (iii) demonstrate the effectiveness of the aforementioned claim. The real and reactive power of AC grid in uncertain atmospheric situation is shown in Fig. 17 (i) and (ii) correspondingly.

### Case Study 3

The electric vehicle (EV) battery, photovoltaic (PV) array, and stand-by battery (SBB) are interconnected via a DC bus, creating a seamless energy transfer system. Throughout the investigation, the authors maintained a constant DC bus voltage of 500 V, which proved beneficial under fluctuating irradiance and atmospheric temperature conditions. As a result of this consistent voltage, both the DC bus and the SBB were able to efficiently provide the required energy to charge the electric vehicle battery efficiently.

**Fig. 18:**
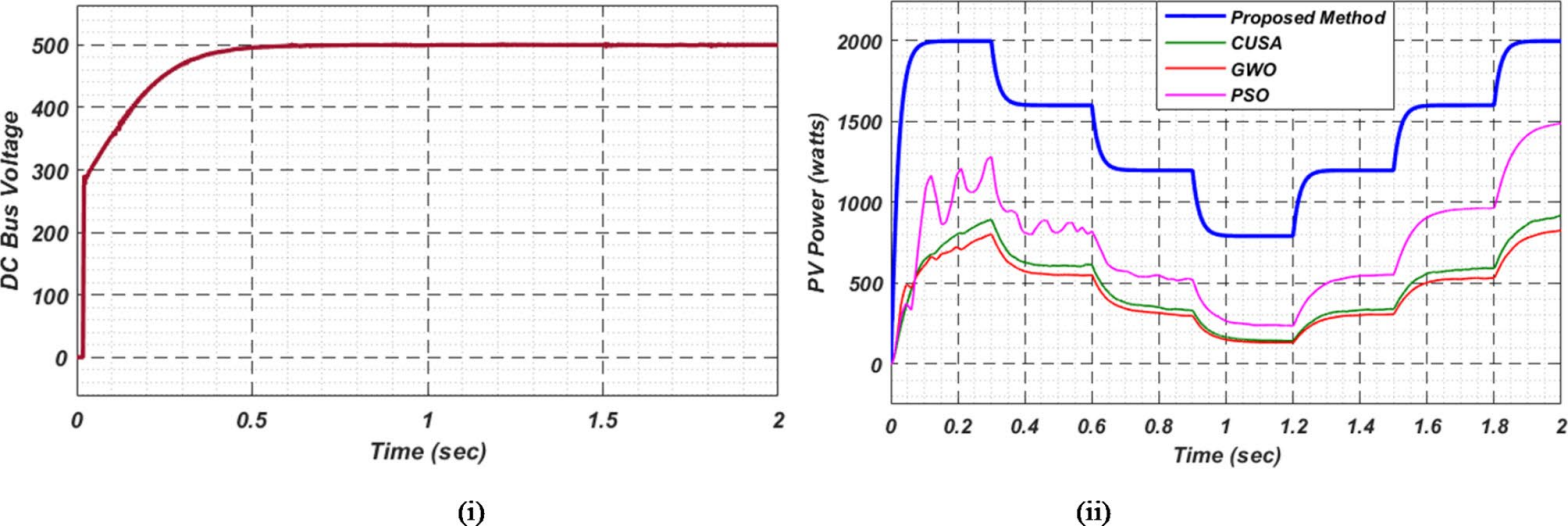
(i) DC bus voltage and (ii) power from PV array for scenario-3.

**Fig. 19:**
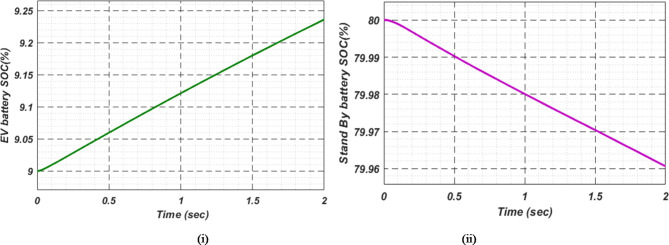
(i) EVs Battery and (ii) Stand-By battery’s state-of-charge (SOC) for case study 3.

In Fig. 18 (i), the stable DC bus voltage is depicted, while Fig. 18 (ii) shows the PV array power output during charging. The SBB reduces its State of Charge as it discharges to provide energy, while the EV battery increases its State of Charge as it charges. In this scenario, the SBB starts with an SoC of 80%, whereas the EV battery starts at a much lower SoC of 9%, as shown in Fig. 19 (i) and (ii). DC buses facilitate an efficient energy management system by allowing the SBB to be discharged while the EV battery is being charged simultaneously.

### Case Study 4

**Fig. 20:**
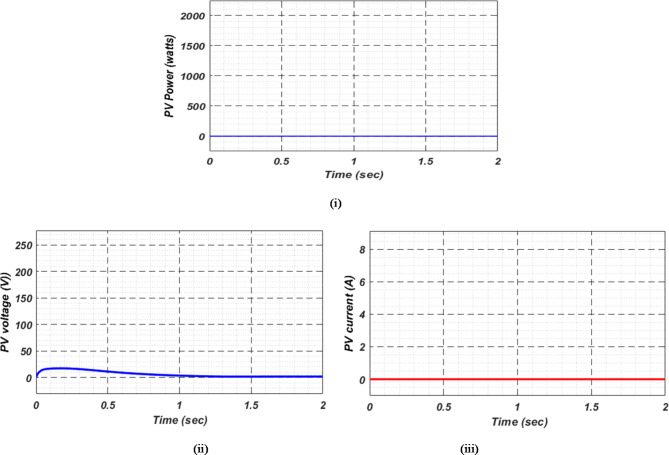
Photovoltaic (i) power (ii) voltage and (iii) current in observation-4.

**Fig. 21:**
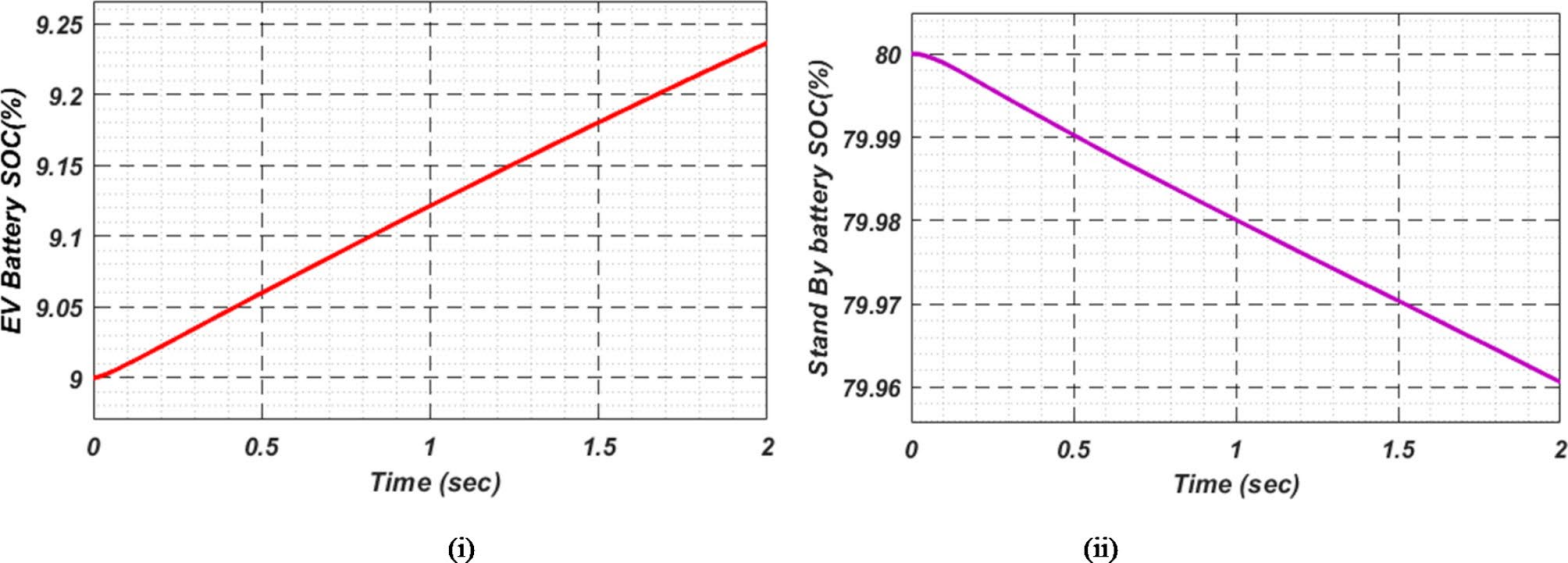
(i) EVs Battery and (ii) SBB’s state-of-charge (SOC) for case study-4.

As a result of insufficient solar irradiance received by the PV panel after dusk, the PV system cannot generate energy. The PV power output of Fig. 20 (i) clearly illustrates this. As illustrated in Fig. 20 (ii) and (iii), the PV voltage and current are almost zero. Thus, since the PV array is not producing power, electric vehicles (EVs) receive their charge solely from their standby batteries (SBBs). The state of charge (SOC) percentages for both the PV and SBB batteries are depicted in Fig. 21 (i) and (ii), providing a clear view of their status.

**Fig. 22:**
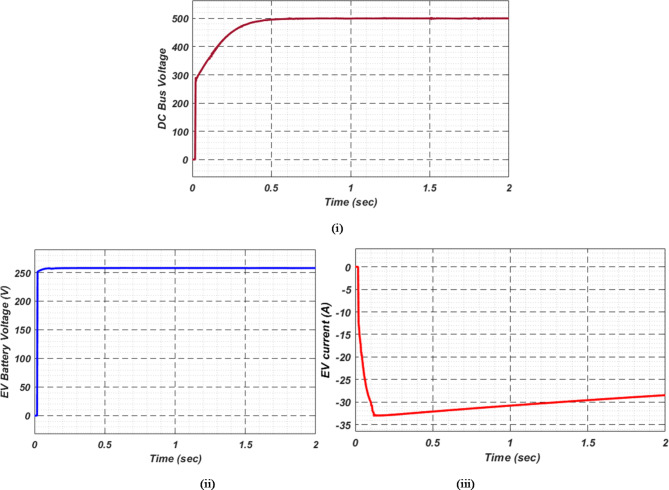
(i) DC bus voltage (ii) EV Battery voltage and (iii) EV current for observation-4.

**Fig. 23:**
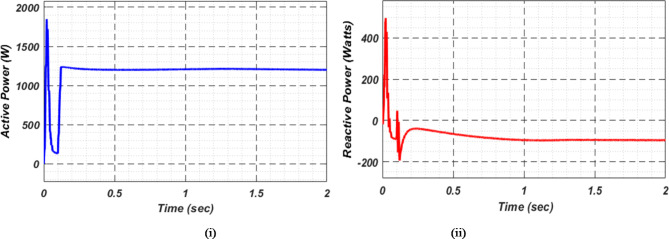
(i) Grid real power (ii) Grid reactive power for scenario-4.

For stable charging of the DC bus, an inverter is connected to the AC grid. To ensure system stability, we maintain a DC bus voltage of 500 volts, while the EV battery voltage is maintained at 250 volts, as shown in Fig. 22 (i) and (ii), respectively. This figure highlights the fact that EV batteries continue to charge regardless of uncertain conditions, as indicated by the negative current flow. Figure 23 (i) and (ii) illustrate the grid’s real and reactive power dynamics. During periods of insufficient PV power generation, the figures also present discrete representations of the voltage and current flowing through the inverter and into the grid. This is to ensure that the DC bus remains powered by the grid during periods when PV power is insufficient.

## Conclusion

A specific sustainable and green energy charging station, together with its simulation and validation under various temperature and irradiance circumstances, are presented in this work. Interconnecting the photovoltaic system, BESS, and electric vehicle charging terminals as a load connected with a single DC bus on the station. Power conversion steps are reduced, and the effectiveness of continuing power supply at the charging terminals increases as benefits accrue from the adoption of a standardised DC bus. Precise and steady power supply is guaranteed by dedicated DC to DC converters employed with photovoltaic arrays, battery energy storage systems, and electric vehicles as loads, which may raise conversion efficacy. In addition to introducing a suggested bidirectional converter for BESS that promises advantages like decreased output ripples, minimised component stress, passive element losses, and built-in redundancy, a boost converter was employed in this study to get the utmost power from a photovoltaic array.

This study examines the importance of electric vehicle charging using a multimodal approach. The suggested power management system is demonstrated and tested using MATLAB/Simulink in a variety of operational modes using a multimodal approach. PV-powered charging stations, in addition to grid support and battery storage systems, are a crucial component of a comprehensive solution. In order to facilitate greater ease of use of charging stations in parking lots and offices, research may be conducted to expand the model to support higher power ratings and capacities. Various scenarios of EV charging should be examined in order to enhance the unpredictable nature of the model. Locations, battery capacity, and charging power levels need to be taken into account.

Solar photovoltaics and electric vehicles require additional research in order to address the evolving penetration scenarios. The development of benchmark studies that take electric vehicle owners’ reactions to grid operator requirements as well as charging expenses into account is still essential to gaining a better understanding of how electric vehicle owners respond to grid operator requirements. As part of this research, vehicle-to-grid (V2G) systems and charging procedures may be developed as a means of balancing the needs of vehicle owners with grid stability. A scale-up architecture is necessary to meet the needs of electric vehicles and higher charging systems. Future developments can be made to the recommended architecture. In addition, EV charging stations with significantly higher capacities and power ratings are available for installation in offices or as part of a larger network. Additional state-of-the-art technologies that could be implement to enhance the sustainability and effectiveness of EV charging terminals include more complex control algorithms and creative energy storage strategies. Furthermore, looking into the addition of renewable energy sources with solar panels could increase the system’s resilience and diversify its energy mix. Keep in mind all this concern, proposed study offers a solid foundation for the creation of charging terminals, promoting future mobility that is sustainable and environmentally responsible.

## Data Availability

Data sets generated during the current study are available from the corresponding author on reasonable request.
